# A Remote Collaborative Care Program for Patients with Depression Living in Rural Areas: Open-Label Trial

**DOI:** 10.2196/jmir.8803

**Published:** 2018-04-30

**Authors:** Graciela Rojas, Viviana Guajardo, Pablo Martínez, Ariel Castro, Rosemarie Fritsch, Markus Moessner, Stephanie Bauer

**Affiliations:** ^1^ Hospital Clínico Universidad de Chile Facultad de Medicina Universidad de Chile Santiago Chile; ^2^ Instituto Milenio para la Investigación en Depresión y Personalidad (MIDAP) Santiago Chile; ^3^ Escuela de Psicología Facultad de Humanidades Universidad de Santiago de Chile Santiago Chile; ^4^ Centro de Innovación en Tecnologías de la Información para Aplicaciones Sociales (CITIAPS) Universidad de Santiago de Chile Santiago Chile; ^5^ Center for Psychotherapy Research University Hospital Heidelberg Heidelberg Germany

**Keywords:** primary health care, depression, telemedicine, rural health care, medically underserved area

## Abstract

**Background:**

In the treatment of depression, primary care teams have an essential role, but they are most effective when inserted into a collaborative care model for disease management. In rural areas, the shortage of specialized mental health resources may hamper management of depressed patients.

**Objective:**

The aim was to test the feasibility, acceptability, and effectiveness of a remote collaborative care program for patients with depression living in rural areas.

**Methods:**

In a nonrandomized, open-label (blinded outcome assessor), two-arm clinical trial, physicians from 15 rural community hospitals recruited 250 patients aged 18 to 70 years with a major depressive episode (DSM-IV criteria). Patients were assigned to the remote collaborative care program (n=111) or to usual care (n=139). The remote collaborative care program used Web-based shared clinical records between rural primary care teams and a specialized/centralized mental health team, telephone monitoring of patients, and remote supervision by psychiatrists through the Web-based shared clinical records and/or telephone. Depressive symptoms, health-related quality of life, service use, and patient satisfaction were measured 3 and 6 months after baseline assessment.

**Results:**

Six-month follow-up assessments were completed by 84.4% (221/250) of patients. The remote collaborative care program achieved higher user satisfaction (odds ratio [OR] 1.94, 95% CI 1.25-3.00) and better treatment adherence rates (OR 1.81, 95% CI 1.02-3.19) at 6 months compared to usual care. There were no statically significant differences in depressive symptoms between the remote collaborative care program and usual care. Significant differences between groups in favor of remote collaborative care program were observed at 3 months for mental health-related quality of life (beta 3.11, 95% CI 0.19-6.02).

**Conclusions:**

Higher rates of treatment adherence in the remote collaborative care program suggest that technology-assisted interventions may help rural primary care teams in the management of depressive patients. Future cost-effectiveness studies are needed.

**Trial Registration:**

Clinicaltrials.gov NCT02200367; https://clinicaltrials.gov/ct2/show/NCT02200367 (Archived by WebCite at http://www.webcitation.org/6xtZ7OijZ)

## Introduction

Depression is a public health problem, a disabling condition that has devastating effects on people’s life and generates high economic costs to society. According to the World Health Organization, depression is the leading cause of disability worldwide and is a major contributor to the overall global burden of disease [1]. Epidemiological studies have demonstrated that 5.5% of the Chilean adult population has suffered from a depressive episode in the last week and that major depression has a lifetime prevalence of 4.7% [2-4]. Studies focusing on primary care services in Santiago, the capital of Chile, have reported a depression prevalence of approximately 30% [5,6].

To reduce the burden of depression in Chile, the Program of Treatment for Depression in Primary Health Care (PTDPHC) was introduced in the early 2000s [7]. This program was later complemented with universal health coverage [8] and the dissemination of clinical practice guidelines as quality standards for primary care clinics [9].

In the PTDPHC, any primary care clinician may refer suspected cases of depression to an on-site physician who can diagnose and initiate treatment [7]. Severe cases are referred to specialized mental health services, and mild to moderate cases may receive a combination of antidepressants, psychosocial interventions, and monitoring visits in primary care, according to severity [7,9].

The PTDPHC was based on a randomized controlled trial in primary care that confirmed the effectiveness and cost-effectiveness of a stepped-care program for the management of depression in resource-limited settings [10,11]. In this trial, the provision of care was highly structured, team-driven, and nonspecialized using available resources in primary care [10].

After a decade of implementation, the PTDPHC has proven to be effective in decreasing depressive symptoms [7]. However, diagnosis inaccuracies, non-guideline-concordant management, and high treatment dropout rates have been identified as pitfalls that reduce PTDPHC performance [6,7]. Moreover, for severe cases, specialized mental health services are unevenly distributed throughout the country [7].

Collaborative care models, which involve primary care teams working in coordination with case managers and mental health specialists [12], have demonstrated to be effective and cost-effective in the treatment of depression [13], leading to implementation efforts to promote its adoption in routine practice [14], thereby being a feasible approach to improve PTDPHC performance.

Although the uneven distribution of specialized mental health services may preclude adoption of these models in rural primary care practices, the use of information and communications technology (ICT) allows mental health specialists to remotely assist primary care teams in underserved locations [15]. These technology-assisted interventions have had positive outcomes in the treatment of depression [16].

These new applications of the collaborative care model may help to reduce the treatment gap in countries with unequal distribution of specialized mental health services, such as Chile [17]. This study reports the feasibility, acceptability, and effectiveness of a remote collaborative care program for patients with depression living in rural areas of Chile.

## Methods

### Study Design

This was a nonrandomized, open-label, two-arm clinical trial with a blinded outcome assessor. Patients in the usual care group were recruited first; 3 months later, patients were enrolled for the intervention.

### Recruitment

This study was carried out at 15 rural community hospitals in the Coquimbo, Bío Bío, and Los Lagos regions in Chile. These hospitals had internet access and a PTDPHC in operation.

Patients aged 18 to 70 years, who had received a new diagnosis of depression, were invited to participate in the study by their primary care physicians at the rural community hospitals and were subsequently interviewed via phone by a blinded research psychologist to assess eligibility. Participants were eligible if they met criteria for a current major depressive episode on the Mini-International Neuropsychiatric Interview (MINI), and were not being treated for depression (ie, patients were not currently attending the PTDPHC). The MINI is a structured psychiatric interview that allows clinicians to diagnose *DSM-IV* mental disorders, and it is available in Spanish [18,19]. Additionally, the MINI was used to assess suicide risk. For those patients with high suicide risk, their primary care physician was informed because physicians are responsible for referral of patients to specialized services.

Full ethical approval was granted by the Universidad de Chile Clinical Hospital Ethics Committee and by each of the Regional Health Services Ethics Committees. Informed consent was obtained after the nature and possible consequences of the study were explained.

### Interventions

Before patients’ recruitment, all primary care teams working at the participating rural community hospitals received 8 hours of training on guideline-concordant depression care based on the national clinical practice guidelines of the PTDPHC [9].

In accordance with the PTDPHC, the following treatment algorithms were used: (1) patients with mild depression received low-intensity psychosocial interventions (ie, guided self-help or physical activity); (2) patients with moderate depression or depression not resolving with initial treatment received selective serotonin reuptake inhibitors as first-line pharmacologic interventions, while gradually intensifying psychosocial interventions and/or pharmacotherapy for those who did not respond; (3) patients with severe depression or who were not responding to previous therapy were offered high-intensity psychosocial interventions, antidepressants, and psychotherapy; and (4) patients with treatment-resistant depression, high suicide risk, psychosis, and/or bipolar disorder were referred to regional specialized mental health services.

#### Remote Collaborative Care Program

A remote collaborative care program is a complex intervention designed to remotely support rural primary care teams to treat depressed patients according to current national clinical practice guidelines for depression [9], maintaining medical decision making within local health services. Rural primary care teams—composed of physicians, psychologists, social workers, midwives, and nurses—provided guideline-concordant, face-to-face care to depressed patients according to the treatment algorithm described in the previous section.

These rural primary care teams were contacted directly via ICT with a centralized and specialized mental health team at the University of Chile Clinical Hospital in Santiago, Chile’s capital city. This mental health team—provided by the study—was composed of six psychiatrists, who interacted with the rural primary care teams via Web-based shared clinical records and/or telephone. The rural primary care teams entered basic clinical data of the locally treated depressed patients in the Web-based shared clinical records, subsequently updating information about their patients’ progress. The information uploaded to this system was reviewed by the mental health team once a week.

In order to complement these data, a call center was administered by a trained nonmedical health professional (midwife) at the university’s facilities, who called the patients to gather information directly from them through a series of structured telephone interviews lasting approximately 30 minutes each. During these interviews, the nonmedical health professional monitored treatment compliance in general (ie, missing appointments, clinical progress, and treatment compliance and medication side effects, if prescribed). The telephone monitoring was carried out once a week during the first month and then every 2 weeks, and the information was available to the rural primary care teams and the mental health team through the Web-based shared clinical record system.

Finally, the mental health team reviewed the data gathered from both sources, once a week, providing remote assistance to the rural primary care teams by entering suggestions into the Web-based shared clinical records and, in special cases, by giving indications to rural primary care clinicians over the telephone.

#### Usual Care

Rural primary care teams in the usual care group were encouraged to follow national clinical practice guidelines of the PTDPHC [9], thus following the same treatment algorithm described in previously.

### Outcomes Assessments

Baseline and follow-up assessments at 3 and 6 months after baseline evaluation were carried out via telephone by a research psychologist at Universidad de Chile Clinical Hospital in Santiago, Chile. The research assessor was blinded to intervention status.

Treatment adherence to antidepressants during the previous 3 months was estimated using the Simplified Medication Adherence Questionnaire [20], and user satisfaction was measured through a depression treatment satisfaction scale ranging from 1=very dissatisfied to 7=very satisfied that was dichotomized. The satisfaction scale has been used previously by members of the research team [10].

Depressive symptoms scores were assessed using the Beck Depression Inventory (BDI-I). The BDI-I is 21-item self-report depression symptom scale with scores ranging from zero to 63. A score of 10 to 19 indicates mild symptoms of depression, a score of 20 to 29 is considered moderate depression, and 30 or higher is considered severe depression. This instrument has good psychometric properties for assessing depressive symptoms [21], and has been previously used in Chile for the evaluation of the PTDPHC [7].

Finally, health-related quality of life was recorded by the 36-item Short Form Survey (SF-36). The SF-36 is a widely used self-report measure of generic health status, providing two summary scores (physical and mental components) ranging from zero to 100 (worse to best possible health status) [22]. The SF-36 has been validated in Chile [23].

### Statistical Analysis

#### Data Analysis

The data input into the electronic platform was extracted to be processed using STATA version 14.0 (StataCorp, College Station, TX, USA). Baseline characteristics were compared between the treatment groups using the chi-square test or Fisher exact test for categorical data and Student *t* test for continuous variables. Mixed-effects logistic regression analyses were performed to test the association between intervention and treatment adherence or user satisfaction at each follow-up, with age, sex, and baseline BDI-I scores as covariates and random effects at the hospital level. The effectiveness of the intervention on depressive symptoms and health-related quality of life was determined using repeated-measures analyses with linear mixed models, with time and intervention group as the independent variables and with random effects at the patient and the hospital level. A *P* value of less than .05 was considered to be statistically significant.

## Results

### Recruitment and Follow-Up

A total of 409 adults were interviewed after local physicians considered they were depressed. After the blinded baseline interview, 159 patients were excluded because they did not meet criteria for depression according to the MINI or were already in treatment, and 250 patients were eligible. In all, 88.4% (221/250) of the sample were followed up at 3 months and 84.8% (212/250) at 6 months after baseline evaluation.

### Sample Characteristics

There were no differences between groups on baseline assessment (Table 1). The majority of the sample were female (216/250, 86.4%), with a mean age of 41.3 (SD 12.6) years, and 47.6% (119/250) were homemakers. The baseline BDI-I score was mean 30.0 (SD 9.0), and 38.0% (95/250) of participants had a high suicide risk according to the MINI.

### Treatment Adherence and User Satisfaction

The remote collaborative care program patients had higher treatment adherence rates than those in the usual care group at 3 months (73/104, 70.2% vs 73/119, 61.3%) and 6 months (60/98, 61.2% vs 55/114, 48.2%). A higher proportion of patients in the intervention group were “very satisfied and satisfied” (74/99, 74.7%) than in the usual care group (66/106, 62.3%) at 3-month follow-up, a trend that was maintained at the 6-month assessment (69/92, 75% vs 55/93, 59.1%). In the mixed-effects analyses, reported in Table 2, significant differences between groups in favor of the remote collaborative care program were observed at 6 months for treatment adherence and user satisfaction.

**Table 1:** Baseline characteristics of participants according to study group.

Variables		Total (N=250)	Usual care (n=139)	Remote collaborative care program (n=111)	*P* value
Sex (female), n (%)		216 (86.4)	117 (84.2)	99 (89.2)	.25^a^
Age, mean (SD)		41.3 (12.6)	41.8 (1.1)	40.6 (1.2)	.45^b^
**Marital status, n (%)**					**.08^a^**
	Single	57 (22.8)	30 (21.6)	27 (24.3)	
	Cohabiting	28 (11.2)	19 (13.7)	9 (8.1)	
	Married	104 (41.6)	49 (35.3)	55 (49.6)	
	Annulled/Divorced	48 (19.2)	33 (23.7)	15 (13.5)	
	Widowed	13 (5.2)	8 (5.8)	5 (4.5)	
**Education, n (%)**					**.71^b^**
	Illiterate	9 (3.6)	5 (3.6)	4 (3.6)	
	Incomplete elementary	60 (24.0)	35 (25.2)	25 (22.5)	
	Complete elementary	35 (14.0)	22 (5.8)	13 (11.7)	
	Complete secondary	79 (31.6)	38 (27.3)	41 (36.9)	
	Incomplete secondary	30 (12.0)	17 (12.2)	13 (11.7)	
	Higher	37 (14.8)	22 (15.8)	15 (13.5)	
**Occupation, n (%)**					**.11^c^**
	Housewife	119 (47.6)	56 (40.3)	63 (56.8)	
	Student	10 (4.0)	6 (4.3)	4 (3.6)	
	Worker	96 (38.4)	62 (44.6)	34 (30.6)	
	Unemployed^d^	21 (8.4)	13 (9.4)	8 (7.21)	
	Retired/pensioner	4 (1.6)	2 (1.4)	2 (1.8)	
Depressive symptoms (BDI-I)^e^, mean (SD)		30.0 (9.0)	30.6 (9.2)	29.4 (8.8)	.29^b^
Prior depressive episode, n (%)		106 (42.4)	62 (44.6)	44 (39.6)	.45^a^
**Suicide risk, n (%)**					**.43^a^**
	None	60 (24.0)	34 (24.5)	26 (23.4)	
	Low	65 (26.0)	33 (23.7)	32 (28.8)	
	Moderate	30 (12.0)	14 (10.0)	16 (14.4)	
	High	95 (38.0)	58 (41.7)	37 (33.3)	

^a^Chi-square test.

^b^Student *t* test.

^c^Fisher exact test.

^d^Not currently working, but seeking work.

^e^BDI-I: Beck Depression Inventory

**Table 2:** Differences in treatment adherence and user satisfaction between the remote collaborative care program and usual care groups.

Variables		Remote collaborative care program, n/N (%)	Usual care, n/N (%)	Estimated intervention effect		*P* value	χ^2^_4_
				OR (95% CI)^a^	AOR (95% CI)^b^		
**Treatment adherence**							
	3 months	73/104 (70.2)	73/119 (61.3)	1.48 (0.85-2.60)	1.53 (0.85-2.75)	.15	13.2
	6 months	60/98 (61.2)	55/114 (48.2)	1.69 (0.98-2.93)	1.81 (1.02-3.19)	.04	12.7
**User satisfaction**							
	3 months	74/99 (74.7)	66/106 (62.3)	1.79 (0.98-3.27)	1.83 (0.99-3.36)	.05	7.0
	6 months	69/92 (75.0)	55/93 (59.1)	2.07 (1.11-3.88)	1.94 (1.25-3.00)	<.001	10.6

^a^Cluster-adjusted odds ratio.

^b^Odds ratio further adjusted by age, sex, and baseline BDI-I scores.

**Table 3:** Differences in depressive symptoms in the remote collaborative care program and usual care groups.

Variables		BDI-I^a^ score, β (95% CI)	*P* value
**Group**			
	Remote collaborative care program	-1.36 (-3.96 to 1.23)	.30
**Time**			
	3 months	-10.16 (-12.14 to -8.18)	<.001
	6 months	-14.00 (-16.00 to -12.01)	<.001
**Group × time**			
	Remote collaborative care program × 3 months	-1.05 (-3.96 to 1.86)	.48
	Remote collaborative care program × 6 months	-0.90 (-3.85 to 2.06)	.55

^a^BDI-I: Beck Depression Inventory

**Table 4:** Differences in health-related quality of life for the remote collaborative care and usual care groups.

Variables		SF-36^a^ Mental component summary		SF-36 Physical component summary	
		β (95% CI)	*P* value	β (95% CI)	*P* value
**Group**					
	Remote collaborative care program	1.12 (-1.09 to 3.33)	.32	0.56 (-1.41 to 2.54)	.58
**Time**					
	3 months	7.61 (5.63 to 9.60)	<.001	–1.62 (-3.28 to 0.04)	.06
	6 months	9.49 (7.49 to 11.49)	<.001	–3.19 (-4.87 to -1.51)	<.001
**Group × time**					
	Remote collaborative care program × 3 months	3.11 (0.19 to 6.02)	.04	–1.51 (-3.95 to 0.93)	.23
	Remote collaborative care program × 6 months	0.77 (-3.73 to 2.19)	.61	0.31 (-2.17 to 2.79)	.81

^a^SF-36: 36-item Short Form Survey

### Depressive Symptoms

The intervention group had a mean decrease in BDI-I score from 29.4 (95% CI 27.7-31.0) to 14.6 (95% CI 12.1-17.0) compared with a decrease from 30.6 (95% CI 29.0-32.1) to 16.8 (95% CI 14.8-18.9) among the usual care group at 6 months. In the linear mixed-effects regression models, using BDI-score data at all time points, the remote collaborative care program had a 1.05 point greater decrease in mean BDI-I from baseline than the usual care group (95% CI -3.96 to 1.86, *P*=.48) at 3 months and a 0.90 point greater decrease from baseline at 6 months (95% CI -3.85 to 2.06, *P*=.55). These differences were not statistically significant (Table 3).

### Health-Related Quality of Life

In the mixed-effects analyses, reported in Table 4, significant differences between groups in favor of remote collaborative care program were observed at 3 months for mental health component summary scores; however, at 6 months these differences were not statistically significant. There were no clear trends over time for remote collaborative care program regarding the physical component summary scores.

## Discussion

### Principal Results

The remote collaborative care program, carried out in 15 community hospitals to support rural primary care teams in the treatment of depressed patients, was feasible and acceptable, achieving higher user satisfaction and better treatment adherence rates at 6 months as compared to usual care. In addition, although depressive symptoms at follow-up did not show significant differences between the remote collaborative care program and usual care, a trend was observed in favor of the intervention group. The remote collaborative care program had a specific effect on mental health-related quality of life at 3 months that disappeared at 6 months, and no differential effect was achieved on physical health-related quality of life at any time point.

### Strengths and Weaknesses of the Study

The remote collaborative care program was an innovative and complex technology-assisted intervention to support rural primary care teams located in different parts of the country in the management of depressive patients.

The rural primary care teams had to face the challenge of treating depression without on-site psychiatrist. Thus, remote collaborative care program may provide timely and appropriate treatment recommendations from online psychiatrist to the local health providers.

Although no significant differences were observed in depressive symptoms between the patients treated in remote collaborative care program and usual care, patients in the remote collaborative care program group achieved higher rates of treatment adherence, suggesting that technology-assisted interventions, such as the one described in this paper, can bring additional benefits to the PTDPHC, afflicted by high rates of treatment dropout, which may hinder its effectiveness [6].

However, results must be viewed in the context of study limitations: it was not a randomized trial and rural primary care teams faced major time limitations and they were not paid for participating in the study.

Furthermore, collaborative care programs are complex because they include several components; therefore, it is necessary to identify the most active components of the programs in order to prioritize them in treatment [24]. In the case of remote collaborative care program, these components were the treatment provided by rural primary care teams (which could include pharmacotherapy and psychotherapy), Web-based supervision by a centralized and specialized mental health team (via Web-based shared clinical record system), and telephone monitoring by a nonmedical health professional. Unfortunately, the study design does not make it possible to determine the contribution of each component to the results obtained.

### Comparison With Prior Work

Increased clinical attention and patient engagement, along with consultation for those patients not achieving improvement, have all been regarded as essential components for the implementation of effective collaborative care programs [25], and the remote collaborative care program integrated all these in a remote fashion with the assistance of ICT.

There is emerging evidence that some of the core elements of collaborative care programs for depression, such as those previously mentioned, can be delivered remotely, providing timely access to mental health care to vulnerable or underserved populations (eg, people living in rural areas). Studies conducted in the United Kingdom and the United States have found that remote collaborative care programs for depression (ie, those complex interventions in which a component is delivered through the use of ICT) are at least as effective as those collaborative care programs delivered face-to-face [26].

A previous study, carried out at primary care centers in Santiago, Chile, proved that a collaborative care program for depression, which included a pharmacological intervention with periodical telephone contact with lay health workers, improved depressive symptoms and health-related quality of life [27]. Although carried out by the same research team in the aforementioned trial, psychiatric consultation was provided face-to-face to physicians in urban—and more resourceful—practices.

### Conclusions

Remote collaborative care programs may support rural primary care teams that do not have the possibility to collaborate with an on-site psychiatrist by providing an acceptable and highly satisfactory intervention for depressed adults and timely advice to primary care teams working in distant parts of a developing Latin American country. Future studies must evaluate treatment process outcomes in a more detailed manner, taking into account the acceptability of these interventions among teams, as well as their cost-effectiveness. Studies of this type should assess changes in users’ symptomatology and functionality, and the direct costs of implementing these kinds of programs and the indirect costs, such as the variation in patient referrals from distant areas to specialized centers.

## Abbreviations

BDI-I: Beck Depression Inventory
ICT: information and communications technology
MINI: Mini-International Neuropsychiatric Interview
PTDPHC: Program of Treatment for Depression in Primary Health Care
SF-36: 36-item Short Form Survey
